# Gut Microbiota Dysbiosis Induced by Intracerebral Hemorrhage Aggravates Neuroinflammation in Mice

**DOI:** 10.3389/fmicb.2021.647304

**Published:** 2021-05-06

**Authors:** Xiaobo Yu, Guoyang Zhou, Bo Shao, Hang Zhou, Chaoran Xu, Feng Yan, Lin Wang, Gao Chen, Jianru Li, Xiongjie Fu

**Affiliations:** ^1^Department of Neurosurgery, Second Affiliated Hospital, School of Medicine, Zhejiang University, Hangzhou, China; ^2^Department of Neurosurgery, The First People’s Hospital of Wenling, Wenling, China

**Keywords:** intracerebral hemorrhage, neuroinflammation, gut microbiota, dysbiosis, T cell

## Abstract

Intracerebral hemorrhage (ICH) induces a strong hematoma-related neuroinflammatory reaction and alters peripheral immune homeostasis. Recent research has found that gut microbiota plays a role in neurodegeneration and autoimmune diseases by regulating immune homeostasis and neuroinflammation. Therefore, we investigated the relationship between ICH, microbiota alteration, and immune responses after hematoma-induced acute brain injury. In our study, we used a mouse model of ICH, and 16S ribosomal RNA sequencing showed that ICH causes gut microbiota dysbiosis, which in turn affects ICH outcome through immune-mediated mechanisms. There was prominent reduced species diversity and microbiota overgrowth in the dysbiosis induced by ICH, which may reduce intestinal motility and increase gut permeability. In addition, recolonizing ICH mice with a normal health microbiota ameliorates functional deficits and neuroinflammation after ICH. Meanwhile, cell-tracking studies have demonstrated the migration of intestinal lymphocytes to the brain after ICH. In addition, therapeutic transplantation of fecal microbiota improves intestinal barrier damage. These results support the conclusion that the gut microbiome is a target of ICH-induced systemic alteration and is considered to have a substantial impact on ICH outcome.

## Introduction

Hemorrhagic stroke is a common type of stroke with a poor prognosis and high mortality rate (Chang et al., 2018; Zhu et al., 2019). Brain injuries after intracerebral hemorrhage (ICH) involve the following: primary injury induced by hemorrhage and hematoma growth and secondary injury caused by a series of pathologic responses (Zhao et al., 2014; Duan et al., 2016; Zhu et al., 2019). Previous studies have shown that surgical hematoma evacuation may reduce mass effects and hematoma-related brain injury (Wilkinson et al., 2018). However, to date, this method has failed to improve long-term functional outcomes in ICH patients (Sattur and Spiotta, 2020). Therefore, a novel therapeutic target for ICH is imperative.

Previous basic and clinical research has indicated that neuroinflammation contributes to the progression of ICH-induced brain injury (Zhu et al., 2019). Neuroinflammation after ICH is a complex process that is mediated by cellular and molecular components (Wang and Dore, 2007). Previous studies have shown that T cells play a defining role in secondary neuroinflammation after acute brain injury (Tschoe et al., 2020). T cell function has been well characterized in ischemic stroke, but its role in ICH is poorly defined (Shichita et al., 2012). Nonetheless, some studies have shown that pro-inflammatory T cells promote vascular permeability and exacerbate brain injury after ICH *via* the production of inflammatory cytokines (Arumugam et al., 2005). In addition, T cells can communicate with microglia and promote microglial polarization into the M1 phenotype (pro-inflammation), which can exacerbate ICH-induced neuroinflammation (Biswas and Mantovani, 2010). Based on the research mentioned earlier, we assumed that T cells might be a potential target for the treatment of ICH.

Evidence suggests that the gut microbiota is a key regulator of T cell homeostasis and neuroinflammation in nervous system diseases. Recently, rapid development in metagenomics, metatranscriptomics, and meta-proteogenomics has revealed the functional relationship between the gut microbiota and the central nervous system (CNS) function, termed the “gut-brain axis,” which has become an emerging field in neuroscience and neuroimmunology (Berer et al., 2011; Cryan and Dinan, 2012). Gut microbiota play a decisive role in many CNS diseases, including Alzheimer’s disease, Parkinson’s disease, and ischemic stroke. A recent study showed that the regulation of gut microbiota influences neuroinflammation and outcome in an experimental stroke model (Singh et al., 2016). However, the impact of ICH on the gut microbiota composition and the contribution of brain injury-specific microbiota alterations on neuroinflammation and outcome after brain injury are still unknown. Therefore, this study aimed to investigate gut microbiota alterations after ICH and its role in the regulation of neuroinflammation reaction induced by ICH.

In our study, we found that gut microbiota dysbiosis is induced by ICH. In turn, gut microbiota instability causes changes in T cell homeostasis, induction of pro-inflammatory response, and deterioration of outcome. Fecal microbiota transplantation (FMT) to normalize post-hemorrhage dysbiosis is associated with an improved neurobehavioral function and ameliorated neuroinflammation. As mentioned earlier, our results point to a novel and highly complex interplay between the brain and the gut microbiota after acute brain injury, in which the gut microbiota are a target of ICH-mediated pathways resulting in dysbiosis as well as an effector of immune homeostasis with profound impact on ICH outcome.

## Materials and Methods

### Animals

Adult male C57 mice (10–12 weeks old, 22–25 g) of specific pathogen-free grades were purchased from Vital River Laboratory Animal Co., Ltd. (Beijing, China). These mice were provided *ad libitum* with access to food and water and housed under a 12/12-h dark/light cycle in specific pathogen-free conditions (three mice per cage). The mice were randomly assigned to the sham and experimental groups after adaptive feeding. All procedures were performed in accordance with the Guide for the Care and Use of Laboratory Animals of the National Institutes of Health and were approved by the Institutional Ethics Committee of the Second Affiliated Hospital, Zhejiang University of Medicine.

### Study Design

All subjects in this study were blinded to the treatment during the experiments and outcome assessment.

#### Experiment 1

To investigate changes in the gut microbiome after ICH, ICH was induced in five mice by injecting collagenase into the basic ganglia region. Fecal matter was collected pre-ICH and ICH (3 days), then the 16S ribosomal RNA (rRNA) gene sequencing was performed.

#### Experiment 2

Thirty-six mice were randomly assigned into the following two groups to investigate the impaired function of the gastrointestinal trace after ICH: sham (*n* = 15), ICH 3 days (*n* = 15), ICH 7 days (*n* = 3), and ICH 14 days (*n* = 3). The complete gastrointestinal tract was removed from the mice (six per group) to assess gastrointestinal motility. Gut permeability was analyzed by plasma fluorescence measurements (*n* = 6). Change in gut micro-construction after ICH was assessed by hematoxylin and eosin staining (per group *n* = 3).

#### Experiment 3

Twelve mice were randomly divided into three groups to study the temporal dynamics of T cells in the brain after ICH: sham (*n* = 3), ICH 1 day (*n* = 3), ICH 3 days (*n* = 3), ICH 7 days (*n* = 3), and ICH 14 days (*n* = 3). Hemorrhage hemispheres were collected for immunofluorescence and flow cytometry. The corresponding antibodies were used to label and identify the different T cells.

#### Experiment 4

Twelve mice were randomly divided into two groups to study the migration of leukocytes after ICH: sham (*n* = 6) and ICH 3 days (*n* = 6). Peyer′s patches (PPs) labeled by fluorescent cell staining dyes carboxyfluorescein succinimidyl ester (CFSE) were used for flow cytometry (*n* = 3 per group), whereas those labeled with CM-Dil were used for immunofluorescence (*n* = 3 per group).

#### Experiment 5

To investigate the changes in neuroinflammation after FMT in ICH mice, 18 mice were randomly divided into five groups: sham (*n* = 6), vehicle 7 days (*n* = 3), vehicle 14 days (*n* = 3), ICH + FMT 7 days (*n* = 3), and ICH + FMT 14 days (*n* = 3). The ipsilateral basal ganglia region samples of the mice were collected for quantitative real-time polymerase chain reaction (qRT-PCR) analysis.

ICH + FMT group mice were treated with the fecal supernatant (100 μl/day, by gavage) from healthy mice, whereas the vehicle group mice were treated with vehicle [100 μl of phosphate-buffered saline (PBS)/day, by gavage] during the same time.

#### Experiment 6

To investigate the effect of microbiota on neurobehavioral function after ICH, healthy microbiota were transplanted into ICH mice. Sixteen mice were randomly divided into two groups (vehicle and ICH + FMT group) for the subsequent experiments. Bodyweight and neurological scores were evaluated in each group at 3, 7, 14, and 28 days after ICH (*n* = 8).

#### Experiment 7

To investigate the causal relationship between gastrointestinal motility and dysbiosis in the gut microbiota, 12 mice were randomly divided into two groups: sham (*n* = 6) and surgical ileus (*n* = 6). The complete gastrointestinal tract was removed from the mice (three per group) to assess their gastrointestinal motility. Three days after surgery, their fecal matters were collected, and 16S rRNA gene sequencing was performed and compared with the composition of their fecal matter presurgery.

#### Experiment 8

To investigate changes in the gut barrier after FMT in ICH mice, 21 mice were randomly divided into three groups: sham (*n* = 7), vehicle 7 days (*n* = 7), and ICH + FMT 7 days (*n* = 7). Colon samples from the mice were collected for immunofluorescence analysis (*n* = 3 per group). Gut permeability was analyzed by plasma fluorescence measurements (*n* = 4).

### Intracerebral Hemorrhage Mouse Model

The ICH mouse model was performed as described previously (Taylor et al., 2017; Chang et al., 2018). Briefly, mice were anesthetized with pentobarbital sodium (40 mg/kg, 1%) *via* intraperitoneal injection. The 0.05 U type VII collagenase (Clostrid-iumhistolyticum; Sigma-Aldrich) prepared in 0.5-μl saline was stereotactically injected into the right basal ganglia (2.5-mm lateral to the bregma, 3-mm deep at a 5° angle). Throughout the surgery, the rectal temperature was maintained at 37.0 ± 0.5°C. The sham mice received the same treatment, including needle insertion, but without collagenase injection.

### Gut Microbiota Analysis

16S rRNA amplicon sequencing was performed by LC-Bio Technology Company (Hangzhou, Zhejiang, China) to analyze gut microbiota. Fresh feces were collected by abdominal massage to avoid contamination by exogenous bacteria. DNA from mouse feces was isolated by using an E.Z.N.A. ^®^Stool DNA Kit (D4015, Omega, Inc., United States) according to the manufacturer’s instructions. The total amount of DNA in each sample was measured using a Qubit fluorometer (Thermo Fisher, MA, United States). PCR amplification of the 16S rRNA sequence was performed using primer sets specific to V4 regions. Final PCR products were purified using AMPure XT beads (Beckman Coulter Genomics, Danvers, MA, United States) and quantified using Qubit (Invitrogen, United States). The amplicon pools were prepared for sequencing, and the size and quantity of the amplicon library were assessed on an Agilent 2100 Bioanalyzer (Agilent, United States) and with the Library Quantification Kit for Illumina (Kapa Biosciences, Woburn, MA, United States), respectively. The libraries were sequenced using the NovaSeq PE250 platform.

Samples were sequenced on an Illumina NovaSeq platform according to the manufacturer’s recommendations provided by LC-Bio. Paired-end reads were assigned to samples based on their unique barcodes and truncated by cutting off the barcode and primer sequences. Paired-end reads were merged using the FLASH software. Quality filtering of the raw reads was performed under specific filtering conditions to obtain the high-quality clean tags according to the formula (v0.94). Chimeric sequences were filtered using Vsearch software (v2.3.4). After dereplication using DADA2, we obtained a feature table and feature sequence. Alpha diversity and beta diversity were calculated by normalizing the same sequences randomly. Then, according to the SILVA (release 132) classifier, the feature abundance was normalized using the relative abundance of each sample. Alpha diversity was applied to analyzing the complexity of species diversity for a sample through five indices, including Chao 1, Observed species, Goods coverage, Shannon, and Simpson, and all these indices in our samples were calculated with QIIME2. Beta diversity was calculated using QIIME2, and graphs were drawn using the R package. BLAST was used for sequence alignment, and the feature sequences were annotated with the SILVA database for each representative sequence. Other diagrams were implemented using the R package (V3.5.2).

### Gastrointestinal Motility Analysis

The mice received 100 μl of fluorescein isothiocyanate-dextran 70 [70,000 Da fluorescein isothiocyanate (FITC)-dextran, 50 mg/ml, Sigma] in 0.9% saline. One hour after administration, the mice were killed, and the entire intestinal tract from the stomach to the colon was removed, and images were acquired using a chemiluminescence detection system (IVIS spectrum, Perkin Elmer). To quantify gastrointestinal motility, the complete gastrointestinal tract was divided into different segments. Each segment was chopped, the liberated luminal contents were homogenized for 1 min, tissue and coarse particles were removed by centrifugation (300 × *g*, 5 min), and the fluorescence of the supernatant was measured using a fluorescence spectrophotometer (SoftMax^®^ Pro5, Molecular Devices). The value obtained was normalized to that of the blank controls and expressed as the percentage of fluorescence per intestinal segment.

### Intestinal Permeability Assay in Mice

*In vivo* intestinal permeability was assessed by oral gavage of the fluorescein isothiocyanate-dextran 4 (600 mg/kg, 100 mg/ml; 4000 Da FITC-Dextran, Sigma) in mice that had been fasted for 6 h. Blood was collected 2 h after gavage, and plasma was prepared by centrifugation at 2,500 × *g* for 10 min. The fluorescence intensity of undiluted plasma was analyzed using a fluorescence spectrophotometer (SoftMax^®^ Pro5, Molecular Devices) at an excitation wavelength of 485 nm and an emission wavelength of 535 nm. The value obtained was normalized to that of the blank controls and expressed as the percentage of fluorescence per mouse. During this process, the researcher was blinded to the experiment.

### Histopathology of Ileum and Colon

Excised ileum and colon tissues were fixed in 4% paraformaldehyde (PFA) and embedded in paraffin. The blocks were serially cut into 5-μm thick sections and stained with hematoxylin and eosin staining. Histological images were obtained using a microscope (Leica, Mannheim, Germany).

### Tracing Migration of Leukocytes From Peyer’s Patches to the Brain

Twenty-four hours after ICH and sham surgery, cells within PPs were labeled by microinjection of fluorescent cell staining dyes CFSE (25 μM in 2 μl of PBS per PP) or CM-Dil (5 μM in 2 μl of PBS per PP) (Invitrogen) as previously described (Singh et al., 2016). Mice were killed 48 h after cell labeling, and brains were prepared either for flow cytometric analysis (CFSE labeling) or immunofluorescence (CM-Dil labeling).

### Fecal Microbiota Transfer

Fresh fecal pellets were collected from five healthy mice between 9 and 10 am and diluted in ice-cold PBS (120 mg feces/1-ml PBS). Briefly, the stool was steeped in cold PBS for approximately 5 min, homogenized for 10 min, and then centrifuged at 1,000 × *g* at 4° for 10 min. The supernatant was transferred to new tubes and used for transplantation. Recipient mice were orally gavaged with streptomycin (500 mg/ml) in 50-μl sterile PBS for the first 2 days after the ICH. The premise of the antibiotic treatment was to decrease the bacterial load in the recipient mice to reduce competition for the repopulation of microbiota from the donor mice. Three days after ICH, 100 μl of fecal supernatant was gavaged to recipient mice daily for 7 days.

### Assessment of Neurobehavioral Function

Behavioral function assessments were performed by two researchers who were blinded to the experiment. Three tests were used to evaluate neurobehavioral function from 3 to 28 days after ICH as previously described (Hua et al., 2002). For the forelimb placing test, each mouse was held by its torso, allowing the forelimb to hang free. Each forelimb was tested 10 times per mouse, and the percentage of trials in which the mouse placed the appropriate forelimb on the edge of the countertop in response to the vibrissae stimulation was determined. For the cylinder test, mice were placed in a transparent cylinder (diameter: 8 cm; height: 25 cm) and allowed to rear 20 times freely. The location of the first forelimb on the wall was recorded. A score was calculated as follows: (right - left)/(right + left + both), in which a greater positive score indicated more severe left hemiparesis. For the corner turn test, the mice could proceed into a 30° corner and then freely turn either right or left to exit the corner. The choice of direction during 10 trials was recorded, and the percentage of right turns was calculated.

### Quantitative Real-Time Polymerase Chain Reaction

Mice were anesthetized, intracardiac perfusion was performed with 0.1 mol/L cold PBS, and the brain was gently collected and trimmed. Total RNA from the hematoma basal ganglia region was isolated using TRIzol reagent (Invitrogen, Thermo Fisher, MA, United States), according to the manufacturer’s protocol. Complementary DNA was synthesized using a PrimeScript^TM^ RT Master Mix (Takara Bio Inc, Shiga, Japan). qRT-PCR was performed using a standard protocol, with Applied Biosystems Quant Studio^TM^ 5 (Thermo Fisher Scientific, Waltham, MA, United States) and TB Green^TM^ Premix Ex Taq^TM^ (Takara BioInc, Shiga, Japan). The specificity of each reaction was evaluated using melting curve analysis. β-actin was used as an internal control. Each reaction was performed in triplicate, and the change in relative target gene expression normalized to the internal control levels was determined using the 2^–△^^△^^*Ct*^ method (Donato et al., 2020). The sequences of the gene-specific primers (Sangon Biotech, Shanghai, China) are listed in Table 1.

**TABLE 1:** Primers used in RT-PCR.

Primer sequences (5′-3′)		
Gene	Forward	Reverse
IL-17	CCCCTTCACTTTCAGGGTCG	CCCCTTCACTTTCAGGGTCG
Foxp3	AGTCTGCAAGTGGCCTGGTT	TGCTCCAGAGACTGCACCAC
IFN-γ	CTGGAGGAACTGGCAAAAGGATGG	GACGCTTATGTTGTTGCTGATGGC
IL-β	CAACCAACAAGTGATATTCTCCATG	GATCCACACTCTCCAGCTGCA
iNOS	CAAGCACCTTGGAAGAGGAG	AAGGCCAAACACAGCATACC
TNF-α	ATGGCCTCCCTCTCAGTTC	TTGGTGGTTTGCTACGACGTG
β-Actin	AGGCATTGTGATGGACTCCG	AGCTCAGTAACAGTCCGCCTA

*iNOS, inducible nitric oxide synthase; TNF-α, tumor necrosis factor-alpha.*

### Immunofluorescence

As previously described, mice were deeply anesthetized and perfused with 20-ml ice-cold 0.1 mol/l PBS, followed by 4% PFA. The brain was collected and then fixed with 4% PFA overnight and 30% sucrose for 72h at 4°C. Then, the brain samples were cut into 10-μm coronal slices for subsequent experiments. The colon tissues were fixed in 4% PFA overnight and cut into 5-μm thick sections. The tissue sections were washed with PBS and incubated with 10% donkey serum containing 0.3% Triton X-100 for 1 h at room temperature. The sections were then incubated overnight with the following antibodies: CD3 (1:50, Santa Cruz Biotechnology, sc-18843), Occludin (1:50, Santa Cruz Biotechnology, sc-133256), and Claudin (1:50, Santa Cruz Biotechnology, sc-166338) at 4°. Then, the sections were washed with PBS and incubated with Alexa Fluor 488-conjugated donkey anti-Rat immunoglobulin G (IgG) (Invitrogen, A-21208), Alexa Fluor 488-conjugated donkey anti-mouse IgG (Invitrogen, A-21202), and Alexa Fluor Plus 594-conjugated donkey anti-mouse IgG (Invitrogen, A-32744) at 37° for 1 h. Finally, the sections were stained with 4′,6-diamidino-2-phenylindole (Abcam, ab104135) and observed under a fluorescence microscope (Leica, Mannheim, Germany). Three sections were examined per mouse. Each brain section was examined under three fields of vision to acquire the mean number of target cells, and each colon section was examined for mean fluorescence intensity using Image J software (NIH).

### Flow Cytometry

Single-cell suspensions were prepared as described previously (Garcia-Bonilla et al., 2015; Posel et al., 2016). Briefly, mice were anesthetized and perfused with 20 ml of ice-cold 0.9% saline to eliminate blood cells. The brain was dissected, minced with fine scissors, and enzymatically digested in Hanks’ balanced salt solution with Liberase Dispase High (62.5 μg/ml, Roche Diagnostics) and DNase I (50 U/ml, Beyotime, Shanghai, China) for 1 h at 37° with gentle agitation. After the digestion, the tissue samples were triturated and passed through a cell strainer (70 μm). The cells were washed and subjected to 5 ml of 25% Percoll density gradient centrifugation (25 min, 800 × *g*, 46∘). The myelin coat and the supernatant were carefully aspirated, and the cell pellet was preserved at the bottom. The cells were resuspended in 3% fetal bovine serum prepared in a fluorescence-activated cell sorting buffer. After that, the cells were incubated with a mixture of antibodies at 4° for 20 min in the dark for cytometric analysis. The antibodies used in the present study included the following: CD45-Alexa Flour 700 (1:100, eBioscience^TM^, 56-0454-82), CD3-PE (1:100, eBioscience^TM^, 12-0032-82), CD4-APC (1:200, eBioscience^TM^, 17-0042-82), CD8-eFlour450 (1:100, eBioscience^TM^, 48-0081-82), and CD25-Alexa Flour 488 (1:100, eBioscience^TM^, 53-0251-82). For intracellular staining, cells were fixed and permeabilized using Foxp3/Transcription Factor Staining Buffer Set (eBioscience^TM^, 00-5523-00) and stained with Foxp3-PE-Cyanine5 (1:100, eBioscience^TM^, 15-5773-82). After staining was completed, the single cells were analyzed on a CytoFLEX flow cytometer (Beckman Coulter, United States), and the results were analyzed using FlowJo version-10.

### Surgical Ileus Mouse Model

A surgical ileus mouse model was used as previously described (Vilz et al., 2012). Briefly, mice were anesthetized with pentobarbital sodium (40 mg/kg, 1%) *via* intraperitoneal injection. The mice were then fixed on a feedback-regulated heating pad with adhesive tape. After shaving and surgical disinfection, the abdominal cavity was then opened 2 cm in length along the median. Two moist sterile cotton applicators were used to place the small intestine content from the pylorus to the cecum. After surgery, the mouse incision was sutured, and mice were placed on a heating lamp until they recovered from anesthesia.

### Statistical Analysis

All data are presented as mean ± standard error of the mean. Data exhibiting the normal distribution and homogeneity of variance between the two groups were compared using the t-test. Persistent neurological functions were analyzed by two-way repeated-measures ANOVA followed by Tukey’s *post hoc* test. Data with non-normal distribution and unequal variance were compared using the Kruskal–Wallis test and a Dunn–Bonferroni test for *post hoc* comparisons. Statistical significance was set at *P* < 0.05. Statistical analyses were performed using GraphPad Prism 8.0 (GraphPad Prism Software Inc, San Diego, CA, United States) and SPSS 22.0 for Windows (IBM Corp., Armonk, NY, United States).

## Results

### Intracerebral Hemorrhage Induces Gut Microbiota Dysbiosis

**FIGURE 1:**
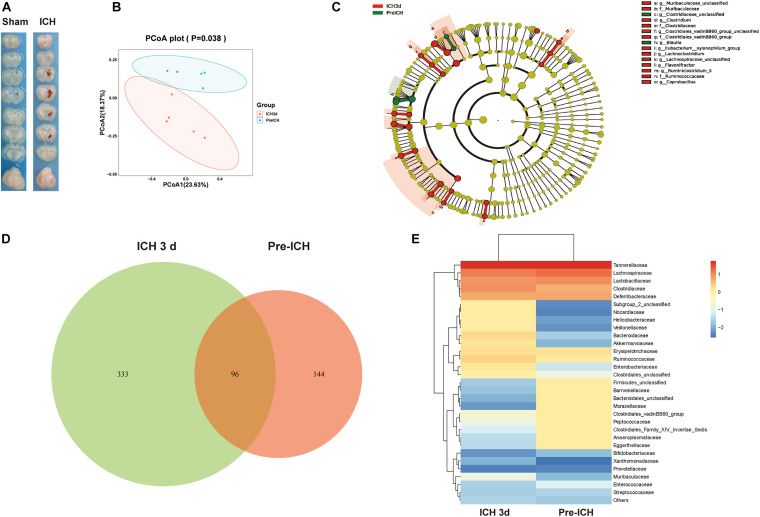
Gut microbiota alterations after intracerebral hemorrhage in mice. **(A)** Images represented of sham and ICH coronal brain sections 3 days after ICH. **(B)** Principal coordinates analysis of the gut microbiota by taxonomic abundance patterns in sham and ICH after 3 days mice (*n* = 5 per group). **(C)** Linear discriminant effect size analysis was performed for indicator taxa analysis identifying features that are statistically different between sham and ICH mice (*n* = 5 per group). **(D)** Venn diagram of the number of differentially features in sham and ICH mice after 3 days (*n* = 5 per group). **(E)** Heat maps of the cluster analysis showing the bacterial composition at the family level in sham and ICH mice after 3 days (*n* = 5 per group).

To investigate whether the ICH is associated with alterations in the intestinal microbiota, we studied the composition of the gut microbiota after ICH in mice using 16S rRNA sequencing of the gut microbiota composition in mice after ICH, with the sham groups showing substantial changes 3 days after ICH (Figures 1A,B). Linear discriminant effect size analysis revealed that the composition of the intestinal microbiota between sham and ICH mice was significantly different, as shown in the cladogram (Figure 1C). The number of different features between sham and ICH 3 days mice was significantly different (Figure 1D). In addition, we have also observed that members of *Firmicutes*, *Barnesiellaceae*, *Bacteriidales*, and *Moraxellaceae* were significantly reduced, whereas *Nocardiaceae*, *Helicobacteraceae*, *Veillonellaceae*, *Bacteroidaceae*, and *Akkermansiaceae* were increased after ICH (Figure 1E).

### Intracerebral Hemorrhage Induced Brain Injury Impaired Gastrointestinal Function and Increased Gut Permeability

**FIGURE 2:**
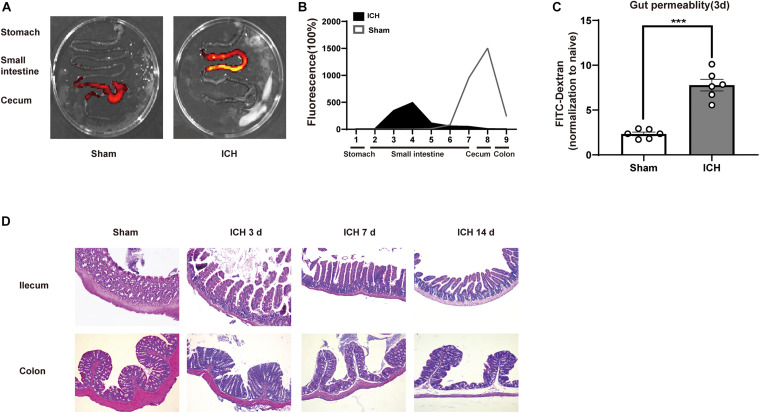
Gastrointestinal dysfunction after ICH. **(A)** Intestinal motility was measured 3 days after sham and ICH surgery. Representative fluorescence images of the complete gastrointestinal tract 1 h after gastric instillation of FITC-dextran showing retention of the fluorescence intensity in intensity segments after ICH. **(B)** Quantification of fluorescent intensity in intestinal segments (*n* = 6 per group). **(C)** Intestinal permeability was measured by oral gavage of FITC-dextran (4 KD) with subsequent analysis of the level plasma of FITC-dextran. There was a significant increase in the ICH mice than the sham group after 3 days. (*n* = 6 per group). **(D)** Intestinal histopathologic changes after ICH 3, 7, and 14 days of ICH. Hematoxylin and eosin staining shows thinning of the layers of epithelium and muscularis mucosae; loss of crypts and glands; edema of the lamina propria; and thickened and shortened villi at the histomorphometric level (*n* = 3 per group). Data are expressed as the mean ± standard error of the mean. ***P < 0.001 *vs*. sham group.

Next, we investigated the mechanisms linking ICH-induced brain injury and dysbiosis of gut microbiota. It has been previously reported that patients with severe brain injury had reduced gastrointestinal motility (Cheng et al., 2018). Consistent with these clinical observations, we detected severe gastrointestinal paralysis after ICH in mice using a gastric fluorescent bolus tracking technique (Figures 2A,B). Furthermore, we found that the intestinal barrier integrity at post-ICH day 3 was increased in treated mice compared with the sham group, as reflected by the increased efflux of circulating FITC-dextran (Figure 2C). Consistently, ICH also causes histological changes characterized by a thinner layer of epithelium and muscularis mucosae, loss of crypts and glands, edema of the lamina propria, and thickened and shortened villi at the histomorphometric level after ICH 3, 7, and 14 days compared with sham mice (Figure 2D).

### Change in T Cells Type After Intracerebral Hemorrhage

**FIGURE 3:**
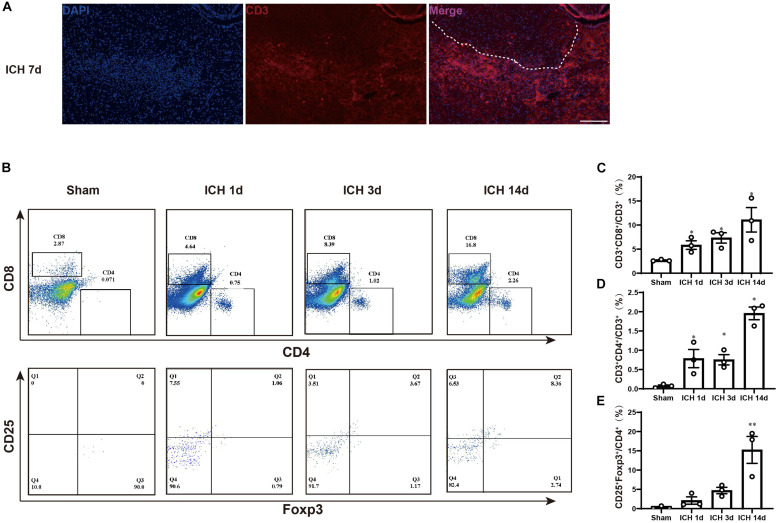
T cell accumulation in the brain after ICH. **(A)** Immunofluorescence staining for CD3 in the hemorrhagic brain on 7 days after ICH. White dotted line indicates the hemorrhagic region. Scare bar = 200 μm. **(B–E)** Flow cytometric analysis of T cells in the hemorrhagic brain on 1, 3, and 14 days after ICH (*n* = 3 per group). Data are expressed as the mean ± SEM. ^∗^*P* < 0.05. ^∗∗^*P* < 0.01. *P* < 0.001 *vs*. sham group.

To determine changes in T cell type after ICH, we first used immunofluorescence to examine whether there was T cell infiltration into the perihematomal region. At 7 days after ICH, we found CD3^+^ cell accumulation around in the perihematomal region (Figure 3A). Then, we used flow cytometry to analyze the different types of T cell changes after ICH. At 1, 3, and 14 days, the experiments were performed, and compared with the sham group mice, the CD4^+^ T cells, CD8^+^ T cells, and regulatory T lymphocytes (Tregs) were increased in the hemorrhagic hemisphere. The trend of the increase was more significant at 14 days after ICH (Figures 3B–E).

The gating strategy for the determination of different T cells is as follows: CD45^+^ cells were represented the leukocytes, the CD3^+^ CD4^+^ population indicates the CD4^+^ T lymphocytes, the CD3^+^ CD8^+^ population represents the CD8^+^ T lymphocytes, and the CD25^+^Foxp3^+^ population is considered a Tregs population.

### T Cells Migrate From the Intestine to the Brain After Intracerebral Hemorrhage

**FIGURE 4:**
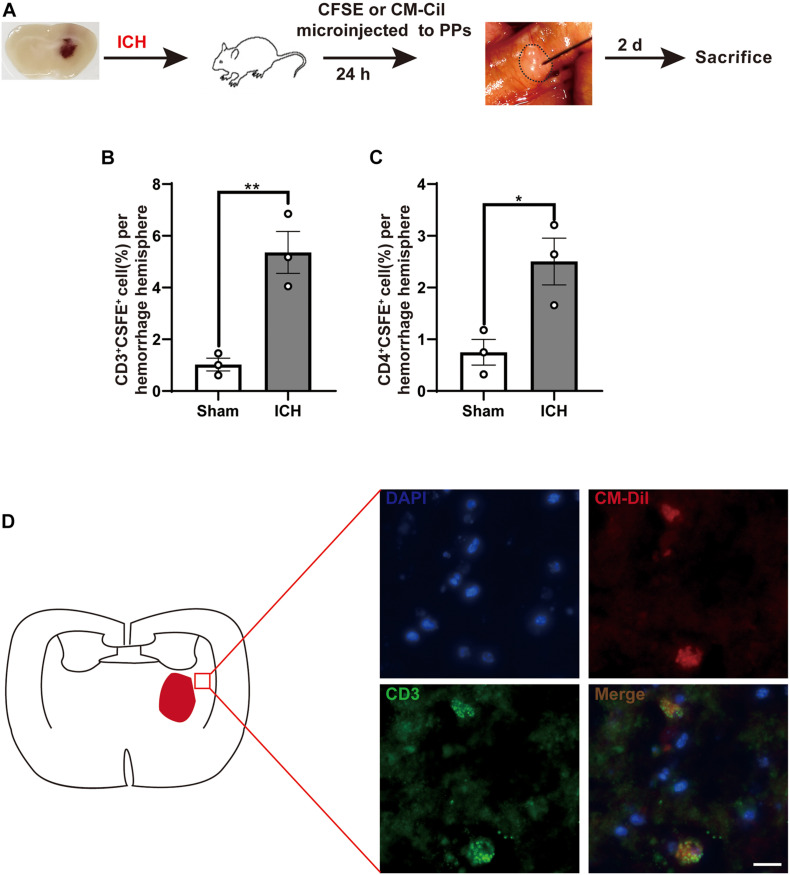
Lymphocytes migrate from the intestine to the brain after ICH. **(A)** Experiment design for tracking the migration of intestinal PP-derived lymphocytes in mice after ICH. CSFE or CM-Cil was microinjected into PPs 24 h after ICH. Two days later, the brain was dissected and analyzed for dye^+^ T cells. **(B,C)** Flow cytometry analysis indicated an increased number of CSFE^+^CD3^+^ and CSFE^+^CD4^+^ in the hemorrhage hemisphere 3 days after ICH compared with sham group (*n* = 3). **(D)** Brain-invading CM-Dil T cells derived from intestinal PPs were identified in the peri-hematoma region and are illustrated as a cumulative map from three mice on one topographical coronal brain section at the bregma level. Data are expressed as the mean ± SEM. *P < 0.05. **P < 0.01 *vs*. sham group. Scale bar = 50 μm.

After ICH, the infiltration of T cells in the perihematomal region plays a key role in ICH-induced neuroinflammation. To investigate potential T cell migration from PPs to the brain, we have used a fluorescent labeling technique by microinjection of CFSE and CM-DiI in all detectable PPs of the mouse intestines after ICH and sham surgery (Figure 4A). Consistent with a previous report, T cells and monocytes fluorescently labeled after microinjection in PPs were detected in the peri-hematoma region 3 days after ICH, consistent with the previously demonstrated kinetics of post-ICH leukocyte invasion. CFSE-positive cells derived from labeled PPs were increased after ICH (Figures 4B,C). We have also confirmed these findings in an independent experiment using CM-DiI as a lipophilic labeling dye and subsequent histological analysis. Here, superimposing localization of T cells from four mice on one coronal brain section, we have detected brain-invading T cells around the hematoma as previously reported (Figure 4D). These results demonstrate that the invasion of many T cells from the intestine to the peri-hematoma region contributed to the ICH-induced neuroinflammation.

### Fecal Microbiota Transplantation Reduces Neuroinflammation After Intracerebral Hemorrhage

**FIGURE 5:**
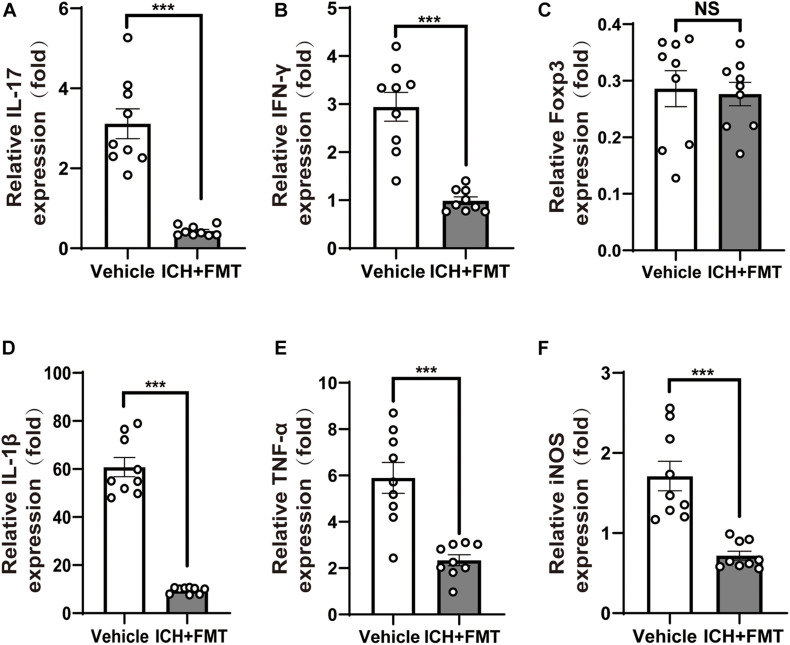
Healthy fecal microbiome transplantation decreases neuroinflammation after ICH. **(A–C)** Recolonizing ICH mice with the gut microbiota obtained from healthy donor mice significantly decreased the gene expression level of IL-17 and IFN-γ compared with recipient of PBS ICH mice 14 days after ICH, but there was no significant change in the gene expression of Fxop3 (*n* = 3 per groups, 3 individual experiments). **(D–F)** Relative gene expression levels of IL-1β, tumor necrosis factor-alpha, and inducible nitric oxide synthase in the hemorrhagic basal ganglia. Recolonization with microbiota from healthy donor mice markedly suppressed IL-1β, tumor necrosis factor-alpha, and inducible nitric oxide synthase expression compared with ICH recipient of PBS ICH mice 14 days after ICH (*n* = 3 per groups, 3 individual experiments). Data are expressed as the mean ± SEM. ***P < 0.001 *vs*. vehicle group.

Given the crucial role of T cells in ICH-induced neuroinflammation, we investigated whether FMT could affect the activity of T cells in the brain after ICH. qRT-PCR was used to examine the expression of the cytokines interleukin 17 (IL-17) and interferon-gamma (IFN-γ) and of the transcription factor Foxp3 as markers of different T_*helper*_ cells in brains 7 and 14 days after ICH. ICH + FMT group mice exhibited massively decreased expression of pro-inflammatory IFN-γ and IL-17 cytokines after ICH for14 days, which are markers of Th1 and Th17 T cells, respectively (*p* < 0.05, Figures 5A,B). However, Foxp3 expression, a marker of Treg cells, did not differ significantly between vehicle and ICH + FMT (Figure 5C).

In addition, we examined the temporal changes in the expression of pro-inflammatory genes in the brain after ICH. The messenger RNA expression levels of pro-inflammatory markers, including IL-1β, inducible nitric oxide synthase, and tumor necrosis factor-alpha, were significantly increased 14 days after ICH. However, this was reversed upon treatment with FMT (*p* < 0.05, Figures 5D–F). Nevertheless, their markers were not significantly different between the two groups after ICH 7 days (Supplementary Figure 1).

### Frequent Fecal Microbiota Transplantation Ameliorated Intracerebral Hemorrhage-Induced Brain Injury

**FIGURE 6:**
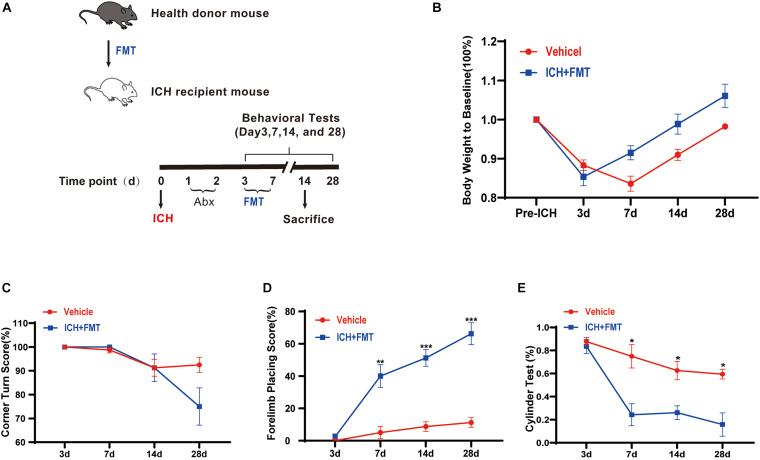
Transplantation of healthy fecal microbiome improves neurobehavioral outcomes after ICH. **(A)** Experimental protocol for fecal microbiota transplant from healthy donor mice to recipient ICH mice. **(B)** Changes in body weight (*n* = 8). **(C–E)** Recolonizing ICH mice with the gut microbiota obtained from healthy donors significantly reduced behavioral performance as assessed by the corner turn test **(C)**, forelimb placing test **(D)**, and cylinder test **(E)** after FMT on 3, 7, 14, and 28 days after ICH. (*n* = 8 per groups, 2 individual experiments). Data are expressed as the mean ± SEM. *P < 0.05. **P < 0.01. ***P < 0.001 *vs*. vehicle group.

To assess the impact of healthy gut microbiota, we have first evaluated the weight changes in vehicle group mice and ICH + FMT mice (Figure 6A). After ICH, mice in our study lost weight (≈13%) by day 3. By day 14, the ICH + FMT group recovered to their pre-ICH body weight (≈1%), whereas the vehicle group did not return to their original body weight (Figure 6B).

Next, we tested the method of FMT as a therapeutic approach to restore the healthy microbiome in animals after ICH. We performed a battery of behavioral tests, including the forelimb placing test, cylinder test, and corner turn test. All groups exhibited the same baseline of neurological function 3 days after ICH. In the forelimb placing test, the ICH + FMT group showed a significant improvement in the percentage of appropriate forelimbs compared with the ICH group on days 7 (5 ± 3.8% *vs*. 40 ± 7.1% in a vehicle), 14 (8.8 ± 3% vs. 51.3 ± 5.2% in a vehicle), and 28 (11.3 ± 3% *vs*. 66.3 ± 6.8% in a vehicle) (*p* 0.05, Figure 6D). In the cylinder test, the FMT group displayed a significant decrease in positive scores (compared to the vehicle group) on days 7 (75 ± 10.2% *vs*. 24.3 ± 9.5% in a vehicle), 14 (62.6 ± 8% *vs*. 26.1 ± 5.9% in a vehicle), and 28 (59.4 ± 4.1% *vs*. 15.8 ± 10.1% in a vehicle) (*p* < 0.05, Figure 6E). Unfortunately, we did not observe a significant difference in the corner turn test result (Figure 6C). Overall, our data indicated that restoration of healthy microbiota ameliorated the ICH-induced neurobehavioral impairment in the subacute phase and later phases.

### Relationship Between Gut Motility Changes and Dysbiosis of Gut Microbiota

**FIGURE 7:**
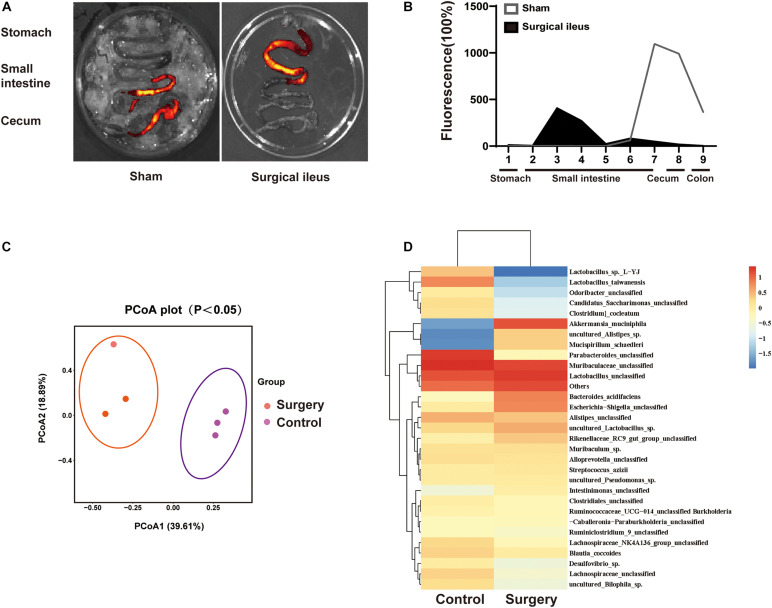
Intestinal motility dysfunction and change in gut microbiota composition after intestinal paralysis. **(A)** Intestinal motility was measure 3 days after control and surgery ileus mice. **(B)** Representative fluorescence images of the complete gastrointestinal tract 1 h after gastric instillation of FITC-dextran showing retention of the fluorescence intensity in intensity segments after intestinal paralysis (*n* = 3 per group). **(C)** Principal coordinates analysis of the gut microbiota composition in control and intestinal paralysis in ICH mice after 3 days (*n* = 3 per group). **(D)** Heat maps of the cluster analysis showing gut microbiota composition in control and intestinal paralysis after 3 days mice (*n* = 3 per group).

To explore the relationship between change in gut motility and gut microbiota dysbiosis. We used a surgical ileus mouse model to mimic the decreased gut motility patterns of ICH mice (Figure 7A,B). The gut microbiota composition was examined by 16S rRNA sequencing after surgery, and the sham mice revealed substantial changes 3 days after surgery (Figure 7C). We have also observed significant changes in the gut microbiota composition (Figure 7D). Our results suggest that the reduced gastrointestinal motility may be one reason for microbiota dysbiosis after ICH, although many factors can influence its composition.

### Frequent Fecal Microbiota Transplantation Restores Intestinal Integrity After Intracerebral Hemorrhage

**FIGURE 8:**
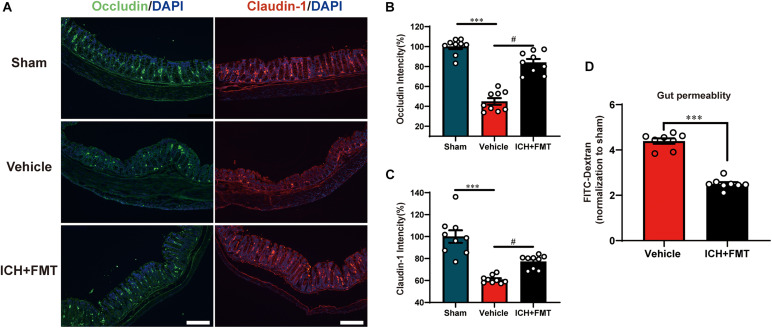
Transplantation of healthy fecal microbiome improves gut intestinal integrity after ICH. **(A)** Representative immunofluorescence staining of the tight junction proteins Occludin and Claudin-1 in sham, vehicle, and ICH + FMT mice colons. **(B,C)** Mean of densities of Occludin and Claudin-1 (*n* = 3 per group, 3 individual experiments). **(D)** Intestinal permeability was improved after FMT treatment for 7 days (*n* = 4 per groups, two individual experiments). Data are expressed as the mean ± SEM. ^∗∗∗^*P* < 0.001 *vs*. sham group. ^ #^*P* < 0.05 *vs*. vehicle group. Scale bar = 200 μm.

This was performed to assess the regulatory effect of FMT on the intestinal barrier after ICH. Compared with the sham group, the mean fluorescence intensity of the tight junction proteins Occludin and Claudin-1 was reduced after ICH but was restored after FMT treatment (Figures 8A–C). FMT treatment also reversed intestinal permeability barrier defects, as determined by the concentration of FITC-dextran in the plasma (Figure 8D).

## Discussion

In this study, we found that gut microbiota play an important role in ICH-induced neuroinflammation in mice. First, we demonstrated that ICH could induce gut microbiota dysbiosis, and this result is in agreement with previous studies in other acute CNS injuries (Benakis et al., 2016). Second, we also found obvious ICH-induced changes in gastrointestinal structure and function. Third, we found that the T cells have undergone dynamic changes after ICH, and in the acute phase of ICH, we found that the intestinal lymphocytes infiltrate into the perihematomal region. Fourth, transplantation of normal microbiota to ICH mice improved neurological outcome, and this effect is related to the attenuation of ICH-induced neuroinflammation. In addition, FMT treatment reduced intestinal barrier damage after ICH. Therefore, we can conclude that the gut microbiota is a key contributor that regulates neuroinflammation after ICH and that regulation of the composition of gut microbiota may be a possible therapeutic target for ICH.

Recent studies have shown that the gut microbiota is involved in the regulation of immune and inflammatory responses in acute and chronic neurological diseases. For instance, spinal cord injury increases intestinal permeability and bacterial translation from the gut, as well as exacerbates neurological impairment (Kigerl et al., 2016). In an ischemic stroke model, a large stroke lesion causes gut dysbiosis of the gut microbiota. In turn, gut microbiota dysbiosis impacts the immunity homeostasis and causes a pro-inflammatory response, leading to the deterioration of neurological stroke outcomes (Benakis et al., 2016; Singh et al., 2016). Patients with Parkinson’s disease have obvious gut microbiota dysbiosis, which leads to an increase in the production of pro-inflammatory cytokines and a decrease in anti-inflammatory bacteria. Thus, gut microbiota dysbiosis is potentially related to Parkinson’s disease state and progression (Liu et al., 2020). Alzheimer’s disease causes gut microbiota imbalance, facilitating the infiltration of peripheral immune cells into the brain parenchyma and enhanced microglial activation, contributing to cognitive decline and amyloid-β burden in a mouse model of Alzheimer’s disease (Kim et al., 2020). However, little is currently known about the changes seen in gut microbiota in ICH-induced brain injury.

Therefore, we explored changes in gut microbiota using a mouse ICH model. Our results show that ICH markedly alters the composition of the gut microbiota, as consistently seen in other CNS diseases (Singh et al., 2016). The diversity of microbiota species was markedly reduced, and gut bacterial changes were observed. As shown in a previous study, patients with ICH have obvious intestinal dysfunction (Cheng et al., 2018). Our results also suggested that ICH can reduce gastrointestinal motility (intestinal paralysis) and increase intestinal permeability in mice. ICH-induced brain injury also alters intestinal structure. In terms of the relationship between gastrointestinal paralysis and gut microbiota dysbiosis, the surgical ileus mouse model also showed significant gut motility decline and changes in gut microbiota composition. Therefore, we hypothesize that ICH-induced intestinal motility may be one reason for gut microbiota dysbiosis. Unfortunately, in our study, we could not declare any definitive causality.

Previous studies have suggested that complex immune and neuroinflammatory cascade responses are key factors in brain injury after ICH. ICH induces the activation of inflammatory cells and the release of cytokines, both of which exacerbate neuroinflammation and influence outcomes (Fu et al., 2021). Neuroinflammation induced by ICH is involved in many cellular and molecular processes (Wang and Dore, 2007). Cellular components include microglia, astrocytes, macrophages, and T cells (Tschoe et al., 2020). In healthy individuals, there are very few T cells in the brain (Hendrix and Nitsch, 2007). Previous studies have shown that cytotoxic T cells infiltrate the perihematomal region as early as 24 h and peaking after 2–7 days (Xue and Del Bigio, 2003). Zhou et al. (2017) found that CD4^+^ T cells were increased 1 day and up to 14 days after ICH. In our study, we also found that T cells can infiltrate into the perihematomal region. The result showed that the CD4^+^ T cells, CD8^+^ T cells, and regulatory T lymphocytes (Tregs) were increased in the hemorrhagic hemisphere 1, 3, and 14 days after ICH. Considering that T cells are involved in ICH-induced neuroinflammation and undergo dynamic change, we hypothesized that T cells are important regulators of neuroinflammation after ICH.

Previous studies have suggested that gut microbiota dysbiosis can exacerbate the neuroinflammation in ischemic stroke through alteration in T cell homeostasis. Restoring gut microbiota homeostasis may have beneficial effects in disease treatment (Singh et al., 2016; Kim et al., 2020). However, whether the gut microbiota regulates neuroinflammation after ICH through T cells is still unclear. Considering the essential role of gut microbiota in the regulation of neuroinflammation, we hypothesized that the gut microbiota is an important regulator of neuroinflammation after ICH. Our results demonstrated that gut microbiota dysbiosis is a key factor in influencing ICH-induced neuroinflammation and, thereby, neurological outcomes in ICH mice. The improved neurological outcomes observe after transplantation of healthy fecal microbiota to ICH mice clearly demonstrated a causal link between gut microbiota dysbiosis and changes in neuroinflammation. After ICH, the cytotoxic T cells were infiltrated into the perihematomal region, as evidenced by the increased levels of pro-inflammatory T cell markers IL-17 and IFN-γ. In line with previous research, our data have indicated that restoring the microbiota homeostasis by FMT significantly decreases the cytotoxic T cell infiltration after ICH, as evidenced by the decreased expression levels of pro-inflammatory cell markers IL-17 and IFN-γ, and can also decrease the levels of pro-inflammation cytokines, IL-1β, tumor necrosis factor-alpha, and inducible nitric oxide synthase. These results suggest that gut microbiota dysbiosis can induce cytotoxic T cell infiltration after ICH. In addition, FMT in ICH mice significantly alleviated ICH-induced secondary brain injury, thus contributing to improved neurological outcomes at 7–28 days after ICH. Moreover, we applied a cell-tracking experiment to intestinal PPs. At 3 days after ICH, the intestinal immune cells can invade into the areas of the brain with hematoma, which is consistent with previous reports of ischemic stroke (Singh et al., 2016). The results also strengthened the observation that gut microbiota regulates neuroinflammation after ICH through T cells.

After ICH, we found significant intestinal barrier damage as inferred from the concentration of FITC-dextran in the plasma. The intestinal barrier consists of three components: surface mucus, the epithelial layer, and immune defenses (Zhang et al., 2021). Tight junction proteins such as Claudin-1 and Occludin in the epithelial layer are essential for gut integrity (Saitou et al., 2000). In our study, we observed that after ICH, the mean fluorescence intensity of the tight junction proteins Occludin and Claudin-1 in the colon was reduced but was restored after FMT treatment. In addition, FMT treatment can decrease the concentration of FITC-dextran in mouse plasma after ICH. This result confirmed that gut microbiota has an impact on the gut barrier and that FMT can reduce intestinal barrier damage. However, the underlying mechanism still needs to be elucidated in future studies.

Although our work has provided evidence that restoring gut microbiota homeostasis attenuates neuroinflammation and improves the outcomes in ICH models in male mice, our study had several limitations. First, we did not elucidate the mechanism underlying the changes in gut microbiota in relation to ICH-induced cerebral immune responses. Further studies are required to elucidate the mechanism of action linking gut microbiota and neuroinflammation. Second, we have explored the effect of gut microbiota in male mice. However, sex and estrogen levels may affect ICH outcomes (Nakamura et al., 2005; Chang et al., 2020). Therefore, further studies are necessary to determine the effect of the gut microbiota in female mice after ICH. Third, age is an important factor that affects the gut microbiota and the functional outcomes of many CNS diseases (Spychala et al., 2018; Lee et al., 2020). However, in our study, we focused on the role of gut microbiota in young mice rather than in older mice. Therefore, more studies are necessary to explore the role and underlying mechanism of gut microbiota in an older mouse model of ICH.

## Conclusion

Our results indicate that significant gut microbiota dysbiosis after ICH contributes to neuroinflammation by affecting T cell homeostasis. In addition, FMT of healthy microbiota in ICH mice attenuates neuroinflammatory injury and improves neurological outcomes. Therefore, restoring gut microbiota homeostasis may minimize ICH-induced brain damage.

## Data Availability Statement

The raw data supporting the conclusions of this article will be made available by the authors, without undue reservation.

## Ethics Statement

The animal study was reviewed and approved by Institutional Ethics Committee of the Second Aliated Hospital, Zhejiang University of Medicine.

## Author Contributions

XF, JL, and GC conceived and designed the study. XY, GZ, HZ, and CX performed the ICH model and PCR. HZ, CX, BS, and FY performed the flow cytometry and immunostaining. LW and XF prepared the figures. XY and GZ analyzed data. XF, XY, and GZ prepared the manuscript draft. GC, LW, and FY wrote the manuscript. All authors contributed to the article and approved the submitted version.

## Conflict of Interest

The authors declare that the research was conducted in the absence of any commercial or financial relationships that could be construed as a potential conflict of interest.
