# Supplementary figures and images for: Gut Microbiota Dysbiosis Induced by Intracerebral Hemorrhage Aggravates Neuroinflammation in Mice
Source: Front Microbiol. 2021 May 6;12:647304. doi: 10.3389/fmicb.2021.647304 (PMC8137318; doi:10.3389/fmicb.2021.647304)

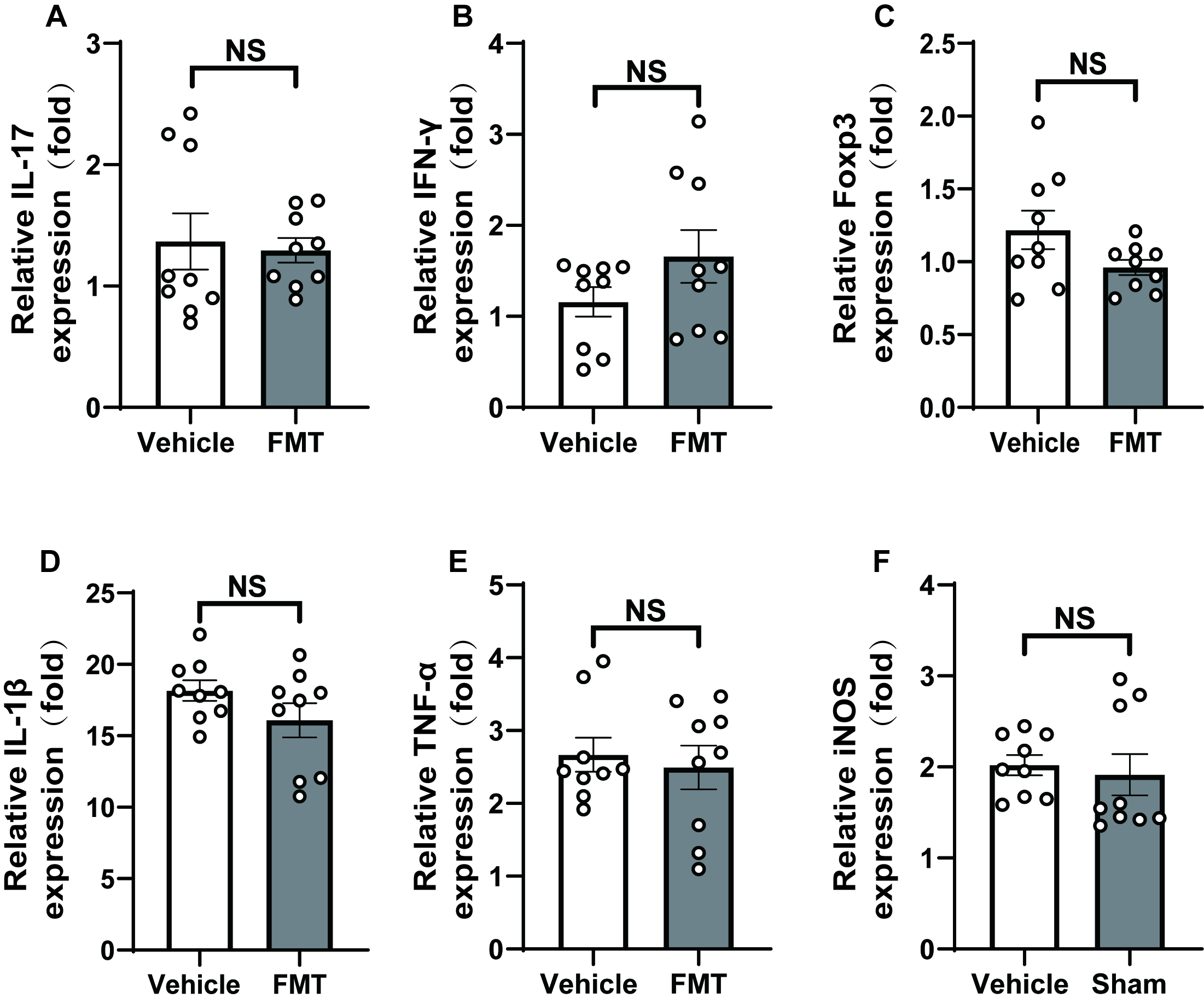

Supplement: Supplementary file 1 [file Image_1.TIF]
